# A Novel Oxazolidinone, Contezolid (MRX-I), Expresses Anti-Mycobacterium abscessus Activity *In Vitro*

**DOI:** 10.1128/AAC.00889-21

**Published:** 2021-10-18

**Authors:** Qi Guo, Liyun Xu, Fusheng Tan, Yongjie Zhang, Junsheng Fan, Xinghai Wang, Zhemin Zhang, Bing Li, Haiqing Chu

**Affiliations:** a Department of Respiratory Medicine, Shanghai Pulmonary Hospital, Tongji University School of Medicine, Shanghai, China; b Tongji University School of Medicine, Shanghai, China; c MicuRx Pharmaceuticals, Inc., Shanghai, China; d Shanghai Key Laboratory of Tuberculosis, Shanghai Pulmonary Hospital, Tongji University School of Medicine, Shanghai, China

**Keywords:** *Mycobacterium abscessu*s, *in vitro*, intracellular, oxazolidinone, contezolid (MRX-I)

## Abstract

An evaluation of the anti-Mycobacterium abscessus activity expressed by a novel oxazolidinone, contezolid (MRX-I), toward 12 reference strains and 194 clinical isolates was conducted. Contezolid was active against M. abscessus *in vitro*, with effects comparable to the anti-M. abscessus effects of linezolid both extracellularly and intracellularly. Contezolid did not antagonize the most frequently used anti-M. abscessus drugs, and preexposure to contezolid did not induce drug resistance. These results provide a novel approach to treating M. abscessus infections.

## INTRODUCTION

Therapeutic options for treating Mycobacterium abscessus infections are extremely limited (1). Oxazolidinones, e.g., linezolid, are recommended by the latest guidelines for treating M. abscessus pulmonary disease (2). However, a high rate of adverse, drug-related reactions (e.g., cytopenia, peripheral neuropathy, and optic neuritis) is a major health concern (3–5).

Contezolid (MRX-I), (*S*)-5-([isoxazol-3-ylamino]methyl)-3-(2,3,5-trifluoro-4-[4-oxo-3,4-dihydropyridin-1(2*H*)-yl]phenyl)oxazolidin-2-one, is a novel oxazolidinone that exhibits antimicrobial effects similar to those of linezolid, i.e., a broad anti-Gram-positive bacterial spectrum when administered orally, and effectiveness in treating methicillin-resistant Staphylococcus aureus, penicillin-resistant Streptococcus pneumoniae, and vancomycin-resistant enterococci (6, 7). It exhibits an improved safety profile, compared to linezolid, and minimal effects with respect to myelosuppression and monoamine oxidase inhibition, two independent adverse events associated with linezolid therapy (6). Importantly, contezolid exhibits anti-Mycobacterium tuberculosis activity both *in vitro* and *in vivo* (8). Therefore, it has potential value for use in long-term combination therapy to treat M. abscessus infections, although supporting data are limited. In the present study, a detailed evaluation of the anti-M. abscessus activity of contezolid was undertaken to determine its potency in treating M. abscessus infections.

### Contezolid is active against M. abscessus.

Antimicrobial susceptibility testing was performed with 12 nontuberculous Mycobacterium reference strains and 194 clinical M. abscessus isolates collected from 182 different patients, according to the Clinical and Laboratory Standards Institute guidelines using the microdilution method (9). Contezolid was active against most nontuberculous Mycobacterium reference strains with the exceptions of Mycobacterium avium and Mycobacterium intracellulare (Table 1). An additional 11 M. intracellulare and 8 M. avium clinical isolates selected at random from ∼200 isolates were also tested and were found to be contezolid insensitive (see Table S1 in the supplemental material). Previous studies conducted by other investigators similarly reported that the majority of M. abscessus isolates were sensitive to linezolid, while >80% of M. avium and M. intracellulare isolates were insensitive (10–15).

**TABLE 1:** MICs of contezolid, linezolid, and tedizolid for 12 nontuberculous Mycobacterium reference strains

Reference strain	MIC (mg/liter) of^*a*^:		
	MRX-I	LZD	TZD
Rapidly growing mycobacteria			
M. abscessus subsp. *abscessus* (ATCC 19977)	16	8	2
M. abscessus subsp. *massiliense* (CIP108297)	16	8	1
Mycobacterium fortuitum (ATCC 6841)	8	4	2
Mycobacterium smegmatis (ATCC 19420)	1	1	1
Mycobacterium peregrinum (ATCC 700686)	1	1	1
Slowly growing mycobacteria			
M. avium (ATCC 25291)	32	16	16
M. intracellulare (ATCC 13950)	64	16	32
Mycobacterium kansasii (ATCC 12478)	1	1	0.125
Mycobacterium gordonae (ATCC 14470)	2	1	0.06
Mycobacterium scrofulaceum (ATCC 19981)	1	0.5	0.125
Mycobacterium marinum (ATCC 927)	4	2	0.5
Mycobacterium xenopi (ATCC 19250)	1	1	0.06

a MRX-I, contezolid; LZD, linezolid; TZD, tedizolid.

Contezolid exhibited anti-M. abscessus activity toward extracellular M. abscessus in culture that was comparable to that of linezolid. The MICs ranged from 0.25 to 64 mg/liter; the MIC_50_ was 16 mg/liter and the MIC_90_ was 32 mg/liter for M. abscessus subsp. *abscessus*, and the MIC_50_ was 8 mg/liter and the MIC_90_ was 32 mg/liter for M. abscessus subsp. *massiliense* (Table 2). The detailed MIC distribution for all clinical isolates is shown in Table S2. Notably, while linezolid and tedizolid exhibited normal MIC distributions, the distribution for contezolid appeared biphasic. A lack of diversity could potentially contribute to this finding, since all the isolates were obtained at a single center. Genotypic and phylogenetic analyses were performed to exclude this possibility, and no duplicate clones were found (see Fig. S1). Therefore, the isolates were genetically diverse, and the biphasic response to contezolid remains to be clarified.

**TABLE 2:** MICs of contezolid, linezolid, and tedizolid for 194 clinical M. abscessus isolates

Antimicrobial agent and species (*n *= 194)^*a*^	MIC_50_ (mg/liter)^*b*^	MIC_90_ (mg/liter)^*b*^	MIC range (mg/liter)	Linezolid susceptibility (%)^*c*^
MRX-I				
M. abscessus subsp*. abscessus* (*n* = 148)	16	32	0.5–64	NA
M. abscessus subsp*. massiliense* (*n* = 46)	8	32	0.25–64	NA
LZD				
M. abscessus subsp*. abscessus* (*n* = 148)	8	32	1–64	54.7^*c*^
M. abscessus subsp*. massiliense* (*n* = 46)	8	32	0.5–64	60.8
TZD				
M. abscessus subsp*. abscessus* (*n* = 148)	1	4	0.125–8	NA
M. abscessus subsp*. massiliense* (*n* = 46)	1	4	0.125–8	NA

a MRX-I, contezolid; LZD, linezolid; TZD, tedizolid.

b MIC_50_ and MIC_90_ are defined as the concentrations at which 50% and 90% of the clinical isolates tested, respectively, were inhibited.

c Sensitivity (MIC of ≤8 mg/liter) and resistance (MIC of ≥32 mg/liter) to linezolid were classified according to Clinical and Laboratory Standards Institute document M24-A2 (9). NA, not applicable.

### Contezolid inhibits the intracellular replication of M. abscessus.

Killing assays were performed according to methods described previously to assess and compare the effects of contezolid and linezolid on the intracellular survival of two reference strains, i.e., ATCC 19977 (M. abscessus subsp*. abscessus*) and CIP108297 (M. abscessus subsp*. massiliense*), and two clinical isolates, i.e., A243 (M. abscessus subsp. *abscessus*) and G71 (M. abscessus subsp. *abscessus*), in primary mouse peritoneal macrophages (16). The cells of both the experimental and control groups were washed three times with warm phosphate-buffered saline to remove the extracellular organisms. Serial dilutions of the supernatants collected after the final wash were cultured on agar plates, and the CFU were counted to ensure that the number of residual extracellular bacteria was negligible.

**FIG 1:**
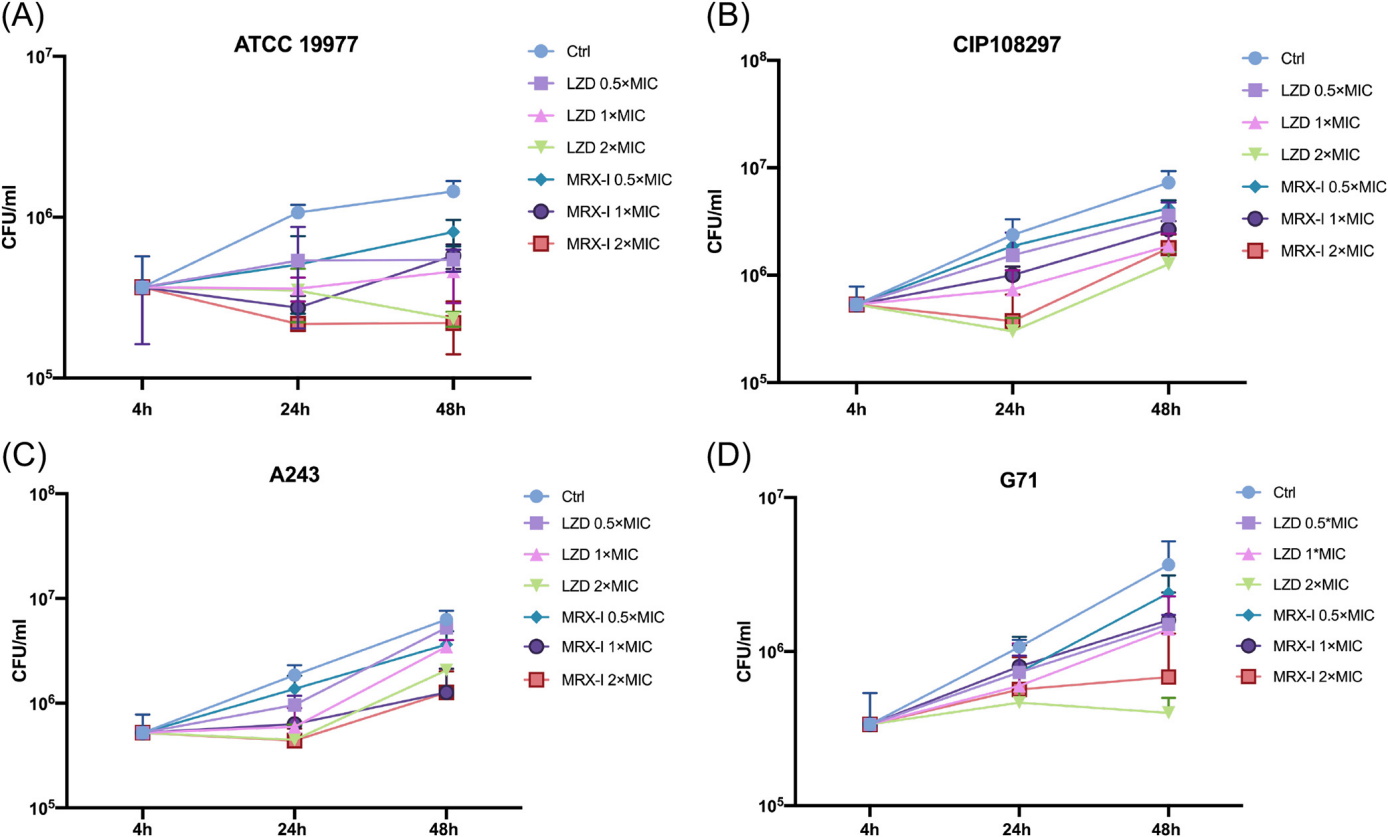
Relative intracellular antimicrobial activities of contezolid and linezolid *in vitro*. (A) M. abscessus subsp. *abscessus* reference strain ATCC 19977; the MIC of linezolid is 8 mg/liter, and the MIC of contezolid is 16 mg/liter. (B) M. abscessus subsp*. massiliense* reference strain CIP108297; the MIC of linezolid is 8 mg/liter, and the MIC of contezolid is 16 mg/liter. (C) M. abscessus subsp. *abscessus* clinical isolate A243; the MICs of both linezolid and contezolid are 2 mg/liter. (D) M. abscessus subsp. *abscessus* clinical isolate G71; the MICs of both linezolid and contezolid are 4 mg/liter. Ctrl, control; MRX-I, contezolid; LZD, linezolid.

Both contezolid and linezolid inhibited the intracellular growth of M. abscessus, relative to the untreated control, for all tested strains; inhibition was dose dependent (Fig. 1). There was no difference in the effects of contezolid and linezolid, indicating comparable intracellular anti-M. abscessus activity. Notably, the structural change in contezolid that results in lower toxicity did not weaken its ability to penetrate cells in our study. Contezolid was equivalent to linezolid and effective in inhibiting both the intracellular and extracellular growth of M. abscessus *in vitro* at the same concentration.

### Contezolid is compatible with drugs most frequently used to treat M. abscessus infections.

M. abscessus infections generally require treatment with multidrug combinations (2, 4). The compatibility between contezolid and eight antimycobacterial drugs that are frequently used therapeutically (i.e., clarithromycin, azithromycin, amikacin, imipenem, cefoxitin, tigecycline, bedaquinoline, and moxifloxacin) was assessed *in vitro* using the broth microdilution chequerboard titration technique and five randomly selected clinical M. abscessus isolates. No antagonism between contezolid and the aforementioned antimycobacterial drugs was evident (see Table S3).

### Preexposure to contezolid does not induce antibiotic resistance in M. abscessus.

The risk of resistance induced by contezolid exposure was determined by preexposing M. abscessus strains (ATCC 19977 and two randomly selected clinical M. abscessus isolates) to contezolid at one-fourth and one-half the MIC and then subsequently quantifying the MICs of contezolid and eight other antibiotics postexposure. The MIC values of contezolid, as well as those of the other eight drugs listed above, did not increase following contezolid preexposure (see Table S4). Huang and coworkers reported similar results, i.e., contezolid exhibited a lower potential than linezolid to induce mutations and resistance in S. aureus (17).

In conclusion, contezolid is active against M. abscessus *in vitro* and is compatible with antibiotics that are most frequently used to treat M. abscessus infections. Therefore, contezolid is a potential candidate to include in novel therapeutic anti-M. abscessus regimens.
