# Supplementary material for: A Novel Oxazolidinone, Contezolid (MRX-I), Expresses Anti-Mycobacterium abscessus Activity In Vitro
Source: Antimicrob Agents Chemother. 2021 Oct 18;65(11):e00889-21. doi: 10.1128/AAC.00889-21 (PMC8522767; doi:10.1128/AAC.00889-21)
Supplement: Supplemental file 1 — Tables S1 to S4 and Fig. S1. Download AAC.00889-21-s0001.pdf, PDF file, 0.5 MB [file aac.00889-21-s0001.pdf]

Supplemental Table 1. MICs of contezolid, linezolid and tedizolid for clinical *M. avium* and *M. intracellulare* isolates[a]

Isolate ID	Species	MIC (mg/L)		
		MRX-I[b]	LZD[b]	TZD[b]
M661	*M. avium*	32	32	16
M688	*M. avium*	16	8	16
M724	*M. avium*	32	64	16
M906	*M. avium*	128	64	32
M855	*M. avium*	8	64	8
M828	*M. avium*	8	16	8
M756	*M. avium*	8	16	8
M733	*M. avium*	128	128	64
M680	*M. intracellulare*	32	32	32
M658	*M. intracellulare*	64	32	32
M805	*M. intracellulare*	32	32	16

M683	*M. intracellulare*	32	16	16
M827	*M. intracellulare*	32	16	32
M660	*M. intracellulare*	32	32	64
M663	*M. intracellulare*	64	32	64
M757	*M. intracellulare*	64	32	64
M2321	*M. intracellulare*	128	32	16
M834	*M. intracellulare*	64	16	32
M709	*M. intracellulare*	64	16	32

[a]One-tenth the total number of each species was selected from the clinical strain library by randomly drawing lots. The selected isolates were obtained from different patients who were confirmed not to be present in the same ward or at the same time.

[b]MRX-I, contezolid; LZD, linezolid; TZD, tedizolid.

Supplemental Table 2. MIC distribution of contezolid, linezolid and tedizolid among 194 clinical, *M. abscessus* isolates

Antimicrobial agent[a]	Subspecies	MICs (mg/L)										MIC_{50}[b]	MIC_{90}[b]
		0.125	0.25	0.5	1	2	4	8	16	32	64		
MRX-1	*abscessus*	0	0	1	32	22	7	10	25	45	6	16	32
	massiliense	0	1	0	7	10	3	6	7	10	2	8	32
	total	0	1	1	39	32	10	16	32	55	8	8	32
LZD	*abscessus*	0	0	0	1	13	31	36	32	25	10	8	32
	massiliense	0	0	1	3	3	6	15	12	5	1	8	32
	total	0	0	1	4	16	37	51	44	30	11	8	32
TZD	*abscessus*	10	21	33	35	32	10	7	0	0	0	1	4
	massiliense	2	7	7	16	7	6	1	0	0	0	1	4
	total	12	28	40	51	39	16	8	0	0	0	1	4

[a]MRX-I, contezolid; LZD, linezolid; TZD, tedizolid.

[b]The MIC_{50} and MIC_{90} are defined as the minimum concentrations at which 50% and 90%, respectively, of the clinical isolates are inhibited.

Supplemental Table 3. Fractional inhibitory concentration index of contezolid in combination with agents commonly used clinically to treat *M. abscessus* infections

Combination	*M. abscessus* isolates (n=5)[a]		Interaction
	average FICI[b]	Range (min-max)	
MRX-I/azithromycin	1.06	0.75-1.5	Indifference
MRX-I/clarithromycin	1.21	0.75-1.5	Indifference
MRX-I/cefoxitin	2.15	1.5-3	Indifference
MRX-I/imipenem	0.75	0.52-1	Indifference
MRX-I/tigecycline	1.4	1-2	Indifference
MRX-I/bedaquinoline	0.9	0.75-1	Indifference
MRX-I/amikacin	1.2	0.75-2	Indifference
MRX-I/ moxifloxacin	1.21	0.75-2.5	Indifference

[a]Randomly selected clinical isolates.

[b]FICI (fractional inhibitory concentration index) = [(MIC of MRX-I in combination/MIC of MRX-I alone) + (MIC of second antibiotic in combination/MIC of second antibiotic alone)]. Synergy = FICI ≤0.5, indifference = FICI between 0.5 and ≤4, antagonism = FICI >4.

Supplemental Table 4. MIC of antibiotics commonly used for *M. abscessus* treatment before and after contezolid exposure[a]

Antibiotic	MIC (mg/L)								
	ATCC 19977			A39			G89		
	Before exposure	After exposure		Before exposure	After exposure		Before exposure	After exposure	
		1/4 MIC	1/2 MIC		1/4 MIC	1/2 MIC		1/4 MIC	1/2 MIC
Contezolid	16	8	8	2	2	2	4	4	4
Clarithromycin	0.5	0.5	0.5	0.06	0.06	0.06	0.5	0.5	0.5
Amikacin	8	8	8	8	8	8	8	8	8
Imipenem	16	16	16	32	32	32	64	64	64
Linezolid	8	8	8	1	1	1	4	4	4
Cefoxitin	16	16	16	64	64	64	32	32	32
Moxifloxacin	4	4	4	4	4	4	16	16	16
Tigecycline	0.5	0.5	0.5	4	4	4	1	1	1
Bedaquiline	0.25	0.25	0.25	0.015	0.015	0.015	0.125	0.125	0.125

[a] MICs of the nine antibiotics listed was determined before and after *M. abscessus* exposure to sub-minimal inhibitory concentrations (1/4 and 1/2 MIC) of contezolid.

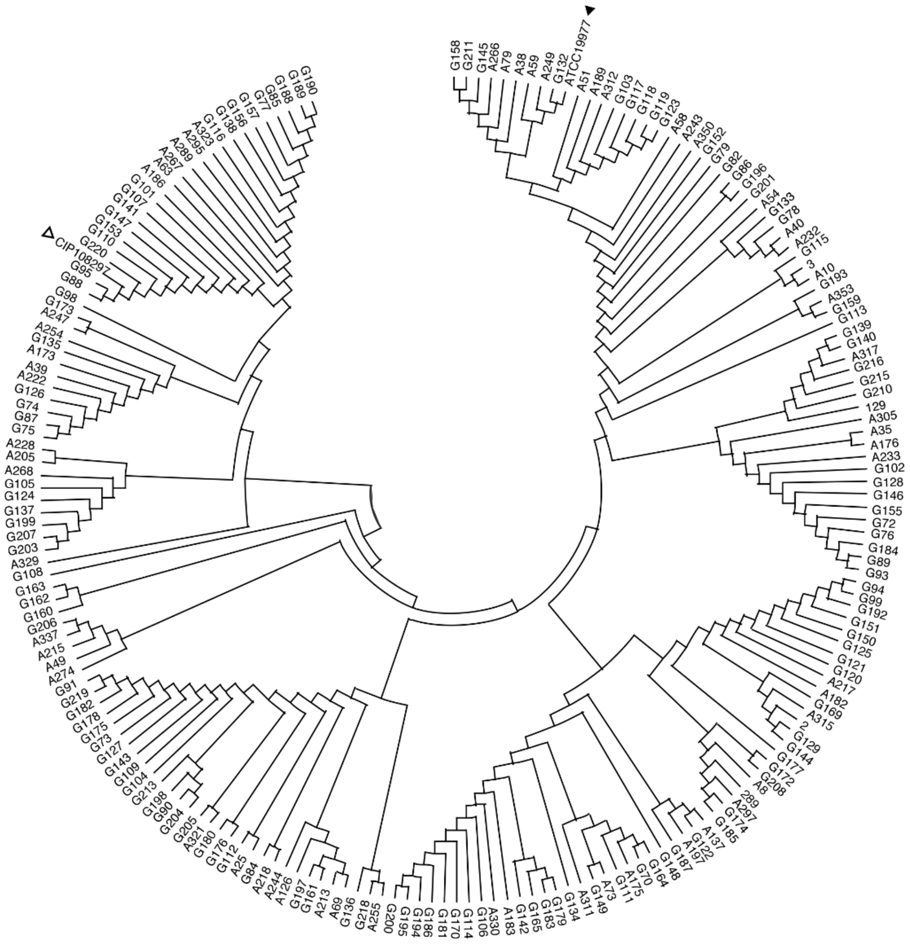

Supplemental Figure 1. Phylogenetic population of 194 *M. abscessus* isolates. The maximum likelihood method and Mega 7.0.26 software were used for analysis. Reference strains ATCC19977 (*M. abscessus* subsp. *abscessus*, solid triangle, ▲) and CIP108297 (*M. abscessus* subsp. *massiliense*, hollow triangle, △) served as controls.
